# High Expression of Complement Component C7 Indicates Poor Prognosis of Breast Cancer and Is Insensitive to Taxane-Anthracycline Chemotherapy

**DOI:** 10.3389/fonc.2021.724250

**Published:** 2021-09-24

**Authors:** Huikun Zhang, Yawen Zhao, Xiaoli Liu, Li Fu, Feng Gu, Yongjie Ma

**Affiliations:** ^1^ Department of Tumor Cell Biology, Tianjin Medical University Cancer Institute and Hospital, National Clinical Research Center for Cancer, Tianjin, China; ^2^ Tianjin’s Clinical Research Center for Cancer, Tianjin Medical University Cancer Institute and Hospital, Tianjin, China; ^3^ Key Laboratory of Cancer Prevention and Therapy, Tianjin, China; ^4^ Key Laboratory of Breast Cancer Prevention and Therapy, Tianjin Medical University, Ministry of Education, Tianjin, China; ^5^ Department of Breast Cancer Pathology and Research Laboratory, Tianjin Medical University Cancer Institute and Hospital, Tianjin, China

**Keywords:** C7, breast cancer, prognosis, TE chemotherapy, bone metastasis

## Abstract

**Background:**

Breast cancer is the most commonly diagnosed cancer worldwide. However, the well-known biomarkers are not enough to meet the needs of precision medicine. Novel targets are desirable and highly valuable for improved patient survival. In this regard, we identified complement component C7 as one of the candidates based on data from the OCOMINE database.

**Methods:**

C7 expression was examined by immunohistochemistry in 331 cases of invasive ductal carcinoma (IDC), 45 cases of ductal carcinoma *in situ* (DCIS), and 52 cases of non-neoplastic tissues adjacent to tumor. Then, C7 expression was further confirmed by Western blot analysis based on IDC specimens and non-neoplastic breast specimens. The relationship between the C7 expression and prognosis of breast cancer patients was analyzed in order to investigate the function of C7 in breast cancer patients. Meanwhile, we also analyzed the relationship between the C7 expression and prognosis of 149 patients treated with conventional TE (taxane and anthracycline)-based chemotherapy. Then, a cohort of patients (22 cases) treated with TE neoadjuvant chemotherapy was used to further confirm the relationship between the C7 expression and TE-based chemosensitivity.

**Results:**

In our present study, we reported for the first time that C7 was an independent prognostic factor of breast cancer and C7 expression of IDC tissues was higher than non-neoplastic tissues adjacent to tumor and DCIS. In a cohort of 331 IDC patients, high expression of C7 indicated poor prognosis especially in the triple negative subtype and luminal B subtype. Furthermore, C7 was also a promoting factor for triple negative subtype patients to develop bone metastasis. Meanwhile, we provided the first evidence that patients with high C7 expression were insensitive to TE (taxane and anthracycline)-based chemotherapy by analyzing a cohort of 149 patients treated with TE-based chemotherapy and another cohort of 22 patients treated with TE neoadjuvant chemotherapy.

**Conclusions:**

In summary, high expression of C7 may promote breast cancer development and might be insensitive to TE-based chemotherapy. Our present study laid a foundation to help clinicians improve the identification of patients for TE-based chemotherapy by C7 in the era of precision medicine.

## Introduction

The incidence and mortality of breast cancer continue to increase and has become the most commonly diagnosed cancer worldwide in 2020 (1–3). However, the well-known biomarkers are not enough to meet the needs of precision medicine. Novel targets are desirable and highly valuable for improved patient survival (4). In this regard, we identified complement component C7 as one of the candidates based on data from the ONCOMINE database.

C7 belonging to the complement system, which is composed of complement natural ingredients, complement control components, and complement receptor, is an important component of the innate immune system and plays a vital role in the coordination of innate and adaptive immune reactions (5, 6). Complement component 7 (C7) is a 93-kDa serum glycoprotein encoded by the *C7* gene. C7 interacts with other terminal complement components (C5b, C6, C8, and C9) to form a membrane attack complex (MAC), which functions as the cytolytic effector unit of the complement system (7). Insertion of the C7 into the cell membrane was identified to be the critical step in the formation of the MAC (8).

Emerging evidence indicated that C7 participated in the progression of several malignancies. It was reported that C7 expression was enhanced in normal tissues, but remarkably reduced in carcinoma tissues of human esophagus, colon, and kidney cancers (9). Additionally, C7 mRNA level expression showed a gradual downward trend in normal, benign, borderline, and malignant ovarian tissues, and C7 expression was negatively related to tumor grade in ovarian cancer patients (10). On the contrary, some researchers hold the opinion that C7 could promote cancer progression. C7 expression was upregulated in ovarian cancer, and knockdown expression of C7 led to a decrease of ovarian cell proliferation (11). Furthermore, significant upregulation of C7 protein in liver tumor-initiating cells was required to maintain the stemness (12).

Until the present, the role of C7 in human breast cancer was unknown. In this study, we identified for the first time that C7 was an independent prognostic factor of breast cancer and its expression was significantly higher in invasive ductal carcinoma (IDC) tissues compared with non-neoplastic tissues adjacent to tumor and ductal carcinoma *in situ* (DCIS). By immunohistochemistry analysis of a large population of 331 IDC cases, we provided the first clinical evidence that a high expression of C7 promoted breast cancer progression. In addition, high expression of C7 was a promoting factor for triple negative subtype patients to develop bone metastasis. Furthermore, we reported for the first time that patients with high C7 expression were insensitive to TE (taxane and anthracycline)-based chemotherapy using a cohort of 149 patients treated with TE-based chemotherapy and another cohort of 22 patients treated with TE neoadjuvant chemotherapy.

## Materials and Methods

### Ethical Statement

All experiments were performed in accordance with relevant guidelines and regulations of the Ethics Committee of Tianjin Medical University Cancer Institute & Hospital. All the patients signed an informed consent for their participation in the study and the use of their biological tissues prior to surgery.

### Clinical Information of Patients

Paraffin-embedded specimens of 331 breast cancer patients with invasive ductal carcinoma (IDC) from 2004 to 2009, the details of IDC patients were shown in **Supplementary Table S1**. A total of 45 patients with breast ductal carcinoma *in situ* (DCIS) and 52 patients with benign lesions, diagnosed between 2008 and 2015, were reviewed and randomly selected from the archives of the Department of Breast Cancer Pathology and Research Laboratory, Tianjin Medical University Cancer Institute & Hospital.

A total of 331 IDC patients were women aging from 28 to 89 years (mean 51.6 years) without preoperative chemotherapy or radiation. The information of subgroups is shown in **Supplementary Table S2**. A total of 319 cases were included for prognostic analyses, excluding cases with no follow-up data (12 cases). These patients were followed up with a median of 71.5 months (5–140 months). Recurrences and distant metastasis were recorded for 20 (6.3%) cases and 58 (18.2%) cases, respectively, and 31 (9.7%) patients died. Among the 319 cases, 236 (74.0%) patients were of the luminal subtype, 34 (10.7%) of the HER2-overexpression subtype, and 49 (15.3%) of the triple negative subtype. A total of 149 (46.7%) patients received TE-based chemotherapy after operation and the rest (170 cases, 53.3%) were treated with other chemotherapies (not TE-based chemotherapy). The details of patients who received non-TE based chemotherapy were the following: 68 cases (CEF: Cyclophosphamide, Epirubicin, and 5-fluorouracil); 59 cases (CMF: Cyclophosphamide, Methotrexate, and 5-fluorouracil); 14 cases (CAF: Cyclophosphamide, Doxorubicin, and 5-fluorouracil); 6 cases (CEF/CMF); and 23 cases (unknown).

### Prognostic Information of Patients

Among the 319 patients with prognostic analyses, 53 patients developed metastasis, recurrence, or death within 5 years; while 165 patients were disease-free over the same 5 years since their diagnosis of breast cancer. A total of 51 patients developed distant metastasis during the follow-up period. In detail, 37 patients developed bone metastasis; 13 patients developed lung metastasis; 13 patients developed liver metastasis; 6 patients developed brain metastasis; and 1 patient developed uterus, kidney, ovarian, and thyroid metastasis, respectively. It was worth noting that multiple organic metastases were noted in 17 patients. Among those 37 IDC patients with bone metastasis, 30 patients were of the luminal subtype, 3 of the HER2-overexpression subtype, and 4 of the triple negative subtype. Among the patients who received TE-based regimens, 28 patients developed metastasis, recurrence, or death within 5 years; while 74 patients were disease-free over the same 5 years.

### Information of 22 Core Needle Biopsy Specimens

We also selected another cohort of patients (22 cases) hospitalized during October 2005 to June 2009. All 22 patients were diagnosed with invasive breast cancer by a 14-gauge core needle biopsy and had completed with preoperative neoadjuvant chemotherapy consisting of 2–8 cycles of TE combined chemotherapy without other local or systemic treatment before surgery. Patients were women 28 to 71 years of age (mean age 56.5 years) and had no other malignant tumors or tumor history. The distribution of clinical involvement showed that all the patients had tumors >2.0 cm. These 22 specimens were collected from each core needle biopsy specimens of primary breast tumor patients before neoadjuvant chemotherapy. All specimens were immediately fixed in 10% normal-buffered formalin and embedded in paraffin and stained for the presence of C7 by immunohistochemistry. The pathological response to neoadjuvant chemotherapy was evaluated after surgical resection of the remaining tumor and assessed according to Miller and Payne histological grading system: grade 1, no change or some alteration to individual malignant cells but no reduction in overall cellularity; grade 2, minor loss (up to 30%) of cancer cells but overall cellularity remains high; grade 3, reduction of 30% to 90% of cancer cells; grade 4, more than 90% loss of cancer cells but small clusters or widely dispersed individual cancer cells remain; and grade 5, no malignant cells identifiable in sections from the site of the tumor consisting of vascular fibroblastic stroma, often containing macrophages; however, DCIS may be present (13). The details of these 22 patients were the following: grade 1 responses (7 cases), grade 2 responses (7 cases), grade 3 responses (5 cases), and grade 4 responses (5 cases). In this study, the 22 patients were divided into two groups: one group was pathological response grade 2 to 4 which was regarded as positive and another group was pathological response grade 1 which was regarded as negative.

### Immunohistochemistry

C7 expression was examined by IHC and the S-P method. In brief, sections (5-μm thick) were dewaxed, hydrated, and heated for 2.5 min for antigen retrieval using citrate buffer. Then, 3% H_2_O_2_ and 10% normal horse serum were applied to reduce endogenous horseradish peroxidase activity and nonspecific staining. Next, sections were incubated with the primary antibody (goat antiserum to human C7, Quidel, USA, A308, 1:3,000) at 4°C overnight. After washing, biotin labeled secondary antibody against goat immunoglobulin was applied for 20 min at room temperature. The slides were rinsed and covered with streptavidin-biotin-peroxidase for 20 min. 3,3’-diaminobenzidinetetrahydrochloride (DAB) was used as the enzyme substrate.

### Evaluation of Staining

Immunostaining of C7 was reviewed by two pathologists in a blinded manner. A consensus judgment was adopted for the intensity score of the tumors based on the strength of C7 expression. Staining intensity was scored as: 0 (-), no staining; 1 (+), definite but weak staining; 2 (++), moderate staining, and 3 (+++), strong staining. Percentage of the positive staining was scored as 0–100. H score was ranged from 0 to 300 by multiplying the intensity and the percentage score. According to the H score of C7, patients were categorized into two groups: low C7 expression (0–119) and high C7 expression (120–300).

### Western Blot

Frozen breast tumor specimens (13 cases) and non-neoplastic breast tissues adjacent to tumor (13 cases) were collected between 2012 and 2015. All patients were women without preoperative chemotherapy or radiation. Tissues were directly lysed in SDS lysis buffer on ice. Equal amounts of cell lysates were loaded and separated by SDS-PAGE, and proteins were transferred onto nitrocellulose membranes and incubated with the primary antibody (goat antiserum to human C7, Quidel, USA, A308, 1:3,000) overnight at 4°C. Membranes were then treated with secondary antibodies. Blots were analyzed by Licor Odyssey infrared imaging.

### Statistical Methods

The GraphPad Prism version 6.0 and the SPSS software Version 19.0 were used for statistical analysis. Mann-Whitney U test and χ^2^ test were performed for group comparisons, and Spearman’s rank correlation test was performed for correlations between two variables. Overall survival (OS) was calculated from pathological diagnosis to the date of last contact or death from breast carcinoma. Progression-free survival (PFS) was defined as the time from surgery to either first disease progression (recurrence or distant metastasis) or cancer-specific death. Survival analyses were performed according to the Kaplan-Meier method. The Cox proportional hazards regression model was performed for the identification of relevant prognostic factors. All tests were two-sided and values of *P* < 0.05 were considered as statistically significant.

## Results

### Bioinformatic and Clinical Analysis Identified C7 Was a Tumor Promoter in Breast Cancer

**Figure 1:**
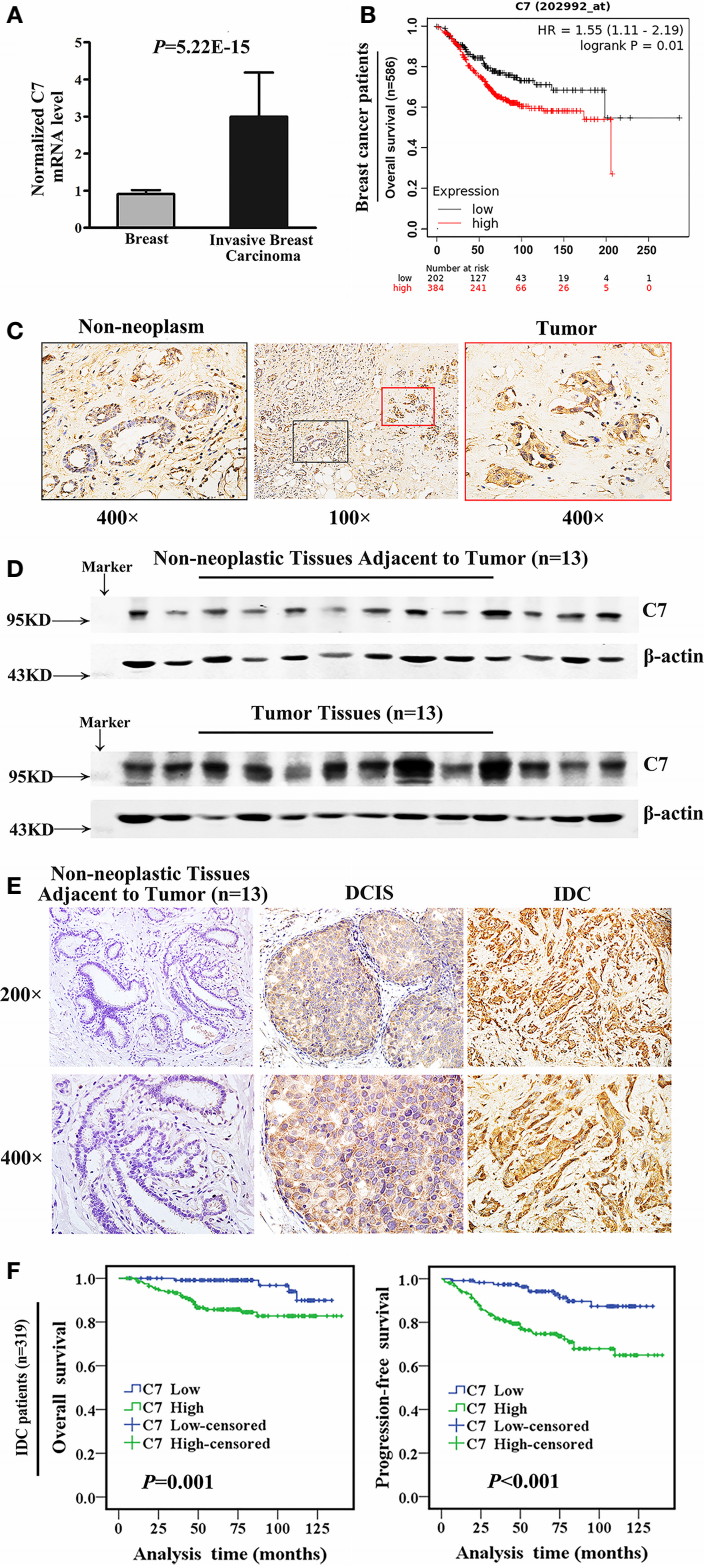
Bioinformatic and clinical analysis identified that C7 was a tumor promoter in breast cancer. **(A)** Normalized C7 mRNA levels were analyzed based on the gene expression profiling data from the ONCOMINE database, including 53 invasive breast carcinoma cases and 6 normal breast samples. **(B)** OS curves of breast cancer patients with C7 mRNA expression in the Kaplan-Meier plotter database. **(C)** C7 expression was shown in non-neoplastic and neoplastic segments. Red rectangle represented tumors and black rectangle represented non-neoplastic tissues adjacent to tumor (magnification 100× and 400×). **(D)** Western blot analysis of C7 expression in breast tumor specimens (IDC, n = 13) and their corresponding non-neoplastic breast tissues adjacent to tumor (n = 13). β-actin was used as a loading control. **(E)** Immunohistochemical staining of C7 in clinical specimens of non-neoplastic breast tissues adjacent to tumor, ductal carcinoma *in situ* (DCIS), and invasive ductal carcinoma (IDC). **(F)** Overall survival (OS) and progression-free survival (PFS) curves of IDC patients (n = 319) (log-rank test).

Firstly, we used a public cancer microarray database, ONCOMINE online (http://www.oncomine.org), to analyze the *C7* mRNA expression level in breast cancer tissues. The data showed that *C7* mRNA expression was upregulated in invasive breast carcinoma (n = 53) compared with normal breast tissues (n = 6) (*P* = 5.22E-15, fold change = 3.018, **Figure 1A**). Next, survival analysis of the Kaplan Meier-plotter database showed that breast cancer patients with a higher *C7* mRNA expression had a shorter overall survival compared with those with a lower *C7* mRNA expression (**Figure 1B**). Then, C7 protein expression was examined by our IHC analysis in 331 cases of IDC, 45 cases of DCIS, and 52 cases of non-neoplastic tissues adjacent to tumor. The intensity of C7 staining was shown in representative images as **Supplementary Figure 1**. In breast tissues, C7 was mainly located in the cytoplasm of epithelial cells of mammary gland ducts. And the immunostaining of C7 was high in tumor cells but much weaker in non-neoplasm in the same section (**Figure 1C**), which was further confirmed by Western blot analysis based on frozen IDC specimens and non-neoplastic breast specimens (**Figure 1D**). In addition, C7 expression of IDC tissues was significantly higher than non-neoplastic tissues adjacent to tumor and DCIS (**Figure 1E** and **Table 1**). A total of 21.2% (11/52) of non-neoplastic tissues adjacent to tumor, 26.7% (12/45) of DCIS, and 63.7% (211/331) of IDC tissue specimens showed a high expression of C7 (χ^2^ = 48.814, *P* < 0.001) (**Table 1**). Moreover, our data showed that IDC patients with a high expression of C7 showed a shorter overall survival (OS) and progression-free survival (PFS) (**Figure 1F**). Altogether, these findings suggested that C7 may play as a tumor promoter in breast cancer.

**Table 1:** C7 expression in different breast tissue specimens.

Histological type	n	C7 score, n (%)		χ^2^	*P*-value^d^
		Low (0–119)	High (120–300)		
Non-neoplastic tissue^a^	52	41 (78.8)	11 (21.2)	48.814	<0.001^***^
DCIS^b^	45	33 (73.3)	12 (26.7)		
IDC^c^	331	120 (36.3)	211 (63.7)		

a Non-neoplastic tissue: non-neoplastic tissues adjacent to tumor.

b DCIS: ductal carcinoma in situ.

c IDC: invasive ductal carcinoma.

d P-value was calculated by Kruskal-Wallis test.

Non-neoplastic tissues vs. IDC: P < 0.001; DCIS vs. IDC: P < 0.001; Non-neoplastic tissues vs. DCIS: P = 0.587.

***P < 0.001.

### High Expression of C7 Indicated Worse Prognosis in Invasive Ductal Carcinoma Patients, Especially in the Triple Negative Subtype and Luminal B Subtype.

**Figure 2:**
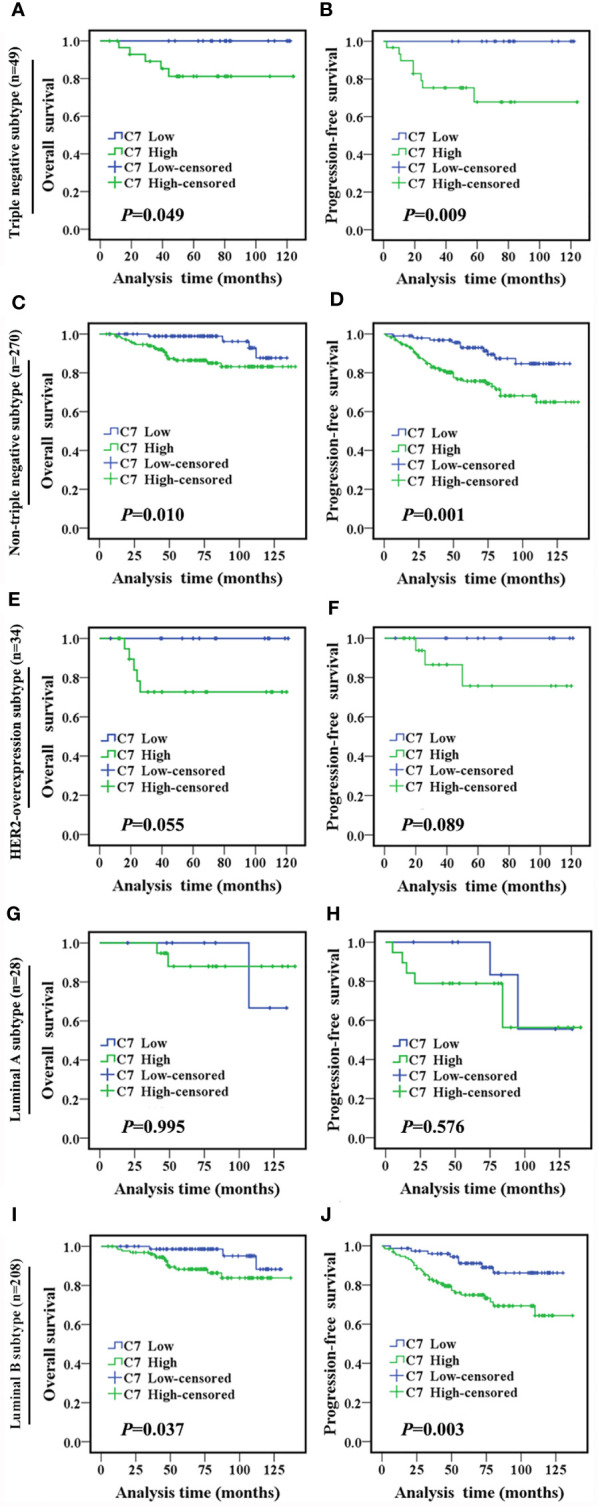
High expression of C7 indicated a shorter survival in IDC patients, especially in the triple negative subtype and luminal B subtype. **(A, B)** OS and PFS curves of patients with triple negative subtype (n = 49). **(C, D)** OS and PFS curves of patients with the non-triple negative subtype (n = 270). **(E, F)** OS and PFS curves of patients with the HER2-overexpression subtype (n = 34). **(G, H)** OS and PFS curves of patients with the luminal A subtype (n=28). **(I, J)** OS and PFS curves of patients with the luminal B subtype (n=208). [**(A–J)**: log-rank test]..

In the following, the total 319 IDC cases were divided into two groups: triple negative subtype (49 cases) and non-triple negative subtype (270 cases). In the triple negative subtype (**Figures 2A, B**) and non-triple negative subtype (**Figures 2C, D**), both the OS and PFS of patients with a high C7 expression were significantly shorter than those with a low C7 expression. Next, Kaplan-Meier analysis was performed in non-triple negative patients with detailed classification. In the HER2-overexpression subtype (n = 34, **Figures 2E, F**) and luminal A subtype (n = 28, **Figures 2G, H**), we found no correlation between C7 and the OS or PFS. However, the results showed that a high expression of C7 indicated a shorter OS (*P* = 0.037, **Figure 2I**) and PFS (*P* = 0.003, **Figure 2J**) in the luminal B subtype (n = 208).

**Figure 3:**
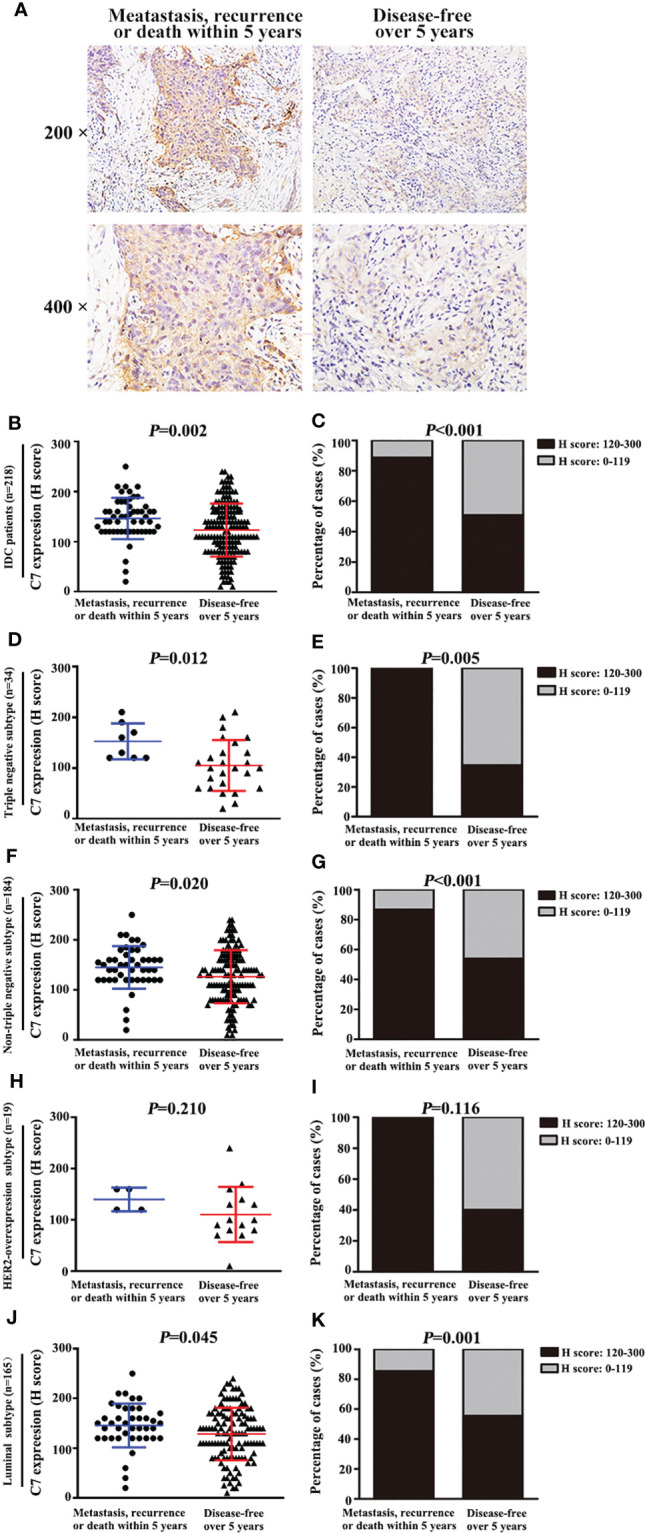
High expression of C7 promoted breast cancer metastasis, recurrence, or death, mainly in the triple negative subtype and luminal B subtype. **(A)** Representative immunohistochemical images of C7 expression in patients who developed metastasis, recurrence, or death within 5 years and patients who were disease-free over 5 years, respectively (magnification 200× and 400×). **(B)** Among 218 IDC patients, C7 expression score in patients who developed metastasis, recurrence, or death within 5 years was higher than those who were disease-free over 5 years (Mann-Whitney U test, *P* = 0.002). **(C)** Among 218 IDC patients, 88.7% (47/53) of patients who developed metastasis, recurrence, or death within 5 years exhibited a high C7 expression, while 50.9% (84/165) of patients who were disease-free over 5 years showed a high C7 expression (*P* < 0.001). **(D, E)** In the triple negative subtype, C7 expression score **(D)** and percentage of high C7 expression **(E)** in patients who developed metastasis, recurrence, or death within 5 years was higher than those who were disease-free over 5 years. **(F, G)** In the non-triple subtype, C7 expression score **(F)** and percentage of high C7 expression **(G)** in patients who developed metastasis, recurrence, or death within 5 years was higher than those who were disease-free over 5 years. **(H, I)** In the HER2-overexpression subtype, there was no difference in the C7 expression score **(H)** or percentage of high C7 expression **(I)** between patients who developed metastasis, recurrence, or death within 5 years and those who were disease-free over 5 years in the luminal B negative subtype, but not in the HER2-overexpression subtype. **(J, K)** In the luminal subtype, C7 expression score **(J)** and percentage of high C7 expression **(K)** in patients who developed metastasis, recurrence, or death within 5 years was higher than those who were disease-free over 5 years. [**(B, D, F, H, J)**: χ^2^ test; **(C, E, G, I, K)**: Mann-Whitney U test].

C7 expression was positively correlated with lymph node metastasis (*r_s_* = 0.162, *P* = 0.003) and distant metastasis (*r_s_* = 0.220, *P* < 0.001) (**Table 2**). Meanwhile, we found that the expression of C7 in patients developing metastasis, recurrence, or death within 5 years (H score: 20.0–250.0, median: 150.0) was higher than those who were disease-free over the same 5 years (H score: 10.0–240.0, median: 120.0, *P* = 0.002, **Figures 3A, B**). Consistently, patients who developed metastasis, recurrence, or death within 5 years exhibited a higher percentage of C7 high expression than those who were disease-free over the same 5 years (**Figure 3C**). Then, the total IDC cases were classified into subgroups according to the molecular subtypes. In the triple negative subtype, the expression of C7 in patients developing metastasis, recurrence, or death within 5 years was higher than those who were disease-free over the same 5 years (**Figure 3D**), and the percentage of high C7 expression in patients who developed metastasis, recurrence, or death within 5 years was significantly higher than those who were disease-free over the same 5 years (**Figure 3E**). Meanwhile, there was a similar trend in the luminal subtype, whereas no such tendency was found in the HER2-overexpression subtype (**Figures 3F–K**). Moreover, in the Cox regression analysis, C7 expression was proved to be an independent prognosis factor for both the OS and PFS in 319 IDC patients (**Table 3**).

**Table 2:** C7 expression and pathological features of IDC patients.

Pathological features	n	C7 score, n (%)		*r*_s_	*P*-value^e^
		Low (0–119)	High (120–300)		
Age, year				0.036	0.514
<50	174	67 (38.5)	107 (61.5)		
≥50	157	55 (35.0)	102 (65.0)		
cTNM stage^a^				0.064	0.249
I	54	21 (38.9)	33 (61.1)		
II	214	83 (38.8)	131 (61.2)		
III-IV	62	18 (29.0)	44 (71.0)		
Histological grade				-0.015	0.779
I	6	1 (16.7)	5 (83.3)		
II	260	97 (37.3)	163 (62.7)		
III	65	24 (36.9)	41 (63.1)		
Tumor size, cm				0.071	0.199
<2	29	12 (41.4)	17 (58.6)		
2-5	250	95 (38.0)	155 (62.0)		
>5	52	15 (28.8)	37 (71.2)		
Lymph node metastasis				0.162	0.003^**^
0	124	55 (44.4)	69 (55.6)		
1–3	82	33 (50.2)	49 (59.8)		
4–9	53	17 (32.1)	36 (67.9)		
>9	72	17 (23.6)	55 (76.4)		
Distant metastasis				0.220	<0.001^***^
No	273	114 (41.8)	159 (58.2)		
Yes	58	8 (13.8)	50 (86.2)		
ER status^b^				0.054	0.328
Negative	127	51 (40.2)	76 (59.8)		
Positive	204	71 (34.8)	133 (65.2)		
PR status^c^				0.009	0.876
Negative	123	46 (37.4)	77 (62.6)		
Positive	208	76 (36.5)	132 (63.5)		
HER2 stauts^d^				0.043	0.431
Negative	219	84 (38.4)	135 (61.6)		
Positive	112	38 (33.9)	74 (66.1)		
Ki-67 status				-0.011	0.846
Negative	45	16 (35.6)	29 (64.4)		
Positive	286	106 (37.1)	180 (62.9)		

a some missing data.

b ER status: estrogen receptor status.

c PR status: progesterone receptor status.

d HER2 status: human epidermal growth factor receptor-2 status.

e P-value was calculated by Spearman’s rank-correlation test.

**P < 0.01, ***P < 0.001.

**Table 3:** Univariate and multivariate analysis for the overall survival (OS) and progression-free survival (PFS) in IDC patients.

Variables	OS (univariate)		OS (multivariate)		PFS (univariate)		PFS (multivariate)	
	HR (95%CI)	*P*	HR (95%CI)	*P*	HR (95%CI)	*P*	HR (95%CI)	*P*
C7 score	4.691 (1.639–13.425)	0.004	3.822 (1.316–11.098)	0.014^*^	3.809 (1.930–7.517)	<0.001	3.188 (1.601–6.350)	0.001^**^
Tumor size	1.293 (0.622–2.668)	0.492	0.718 (0.317–1.624)	0.426	2.114 (1.285–3.478)	0.003	1.270 (0.714–2.257)	0.416
Lymph node metastasis	2.208 (1.567–3.111)	<0.001	2.027 (1.405–2.924)	<0.001	1.718 (1.380–2.138)	<0.001	1.478 (1.153–1.894)	0.002
cTNM	2.045 (1.120–3.734)	0.020	1.260 (0.657–2.418)	0.487	2.296 (1.492–3.535)	<0.001	1.341 (0.803–2.240)	0.261

*P < 0.05, **P < 0.01.

### High Expression of C7 Promoted Breast Cancer Bone Metastasis.

We further explored the relationship between C7 expression and distant metastasis in IDC patients. C7 expression was weakly correlated with bone metastasis in 319 IDC cases (*r_s_* = 0.156, *P* = 0.005), but there was no association between C7 expression and lung metastasis (*r_s_* = 0.092, *P* = 0.100), liver metastasis (*r_s_* = 0.092, *P* = 0.100), or brain metastasis (*r_s_* = 0.106, *P* = 0.058, **Table 4**). Meanwhile, we noticed that the positive association between C7 and bone metastasis might be because there were more events of bone metastasis in breast cancer which made it easier to detect a positive correlation in statistics.

**Table 4:** Relationship between C7 expression and distant metastasis in IDC patients.

Distant metastasis	n	C7 score, n (%)		*r*_s_	*P*-value^a^
		Low (0–119)	High (120–300)		
Bone metastasis				0.156	0.005^**^
No	282	112 (39.7)	170 (60.3)		
Yes	37	6 (16.2)	31 (83.8)		
Lung metastasis				0.092	0.100
No	306	116 (37.9)	190 (62.1)		
Yes	13	2 (15.4)	11 (84.6)		
Liver metastasis				0.092	0.100
No	306	116 (37.9)	190 (62.1)		
Yes	13	2 (15.4)	11 (84.6)		
Brain metastasis				0.106	0.058
No	313	118 (37.7)	295 (62.3)		
Yes	6	0 (0.0)	6 (100)		

a P-value was calculated by Spearman’s rank-correlation test.

**P < 0.01.

**Figure 4:**
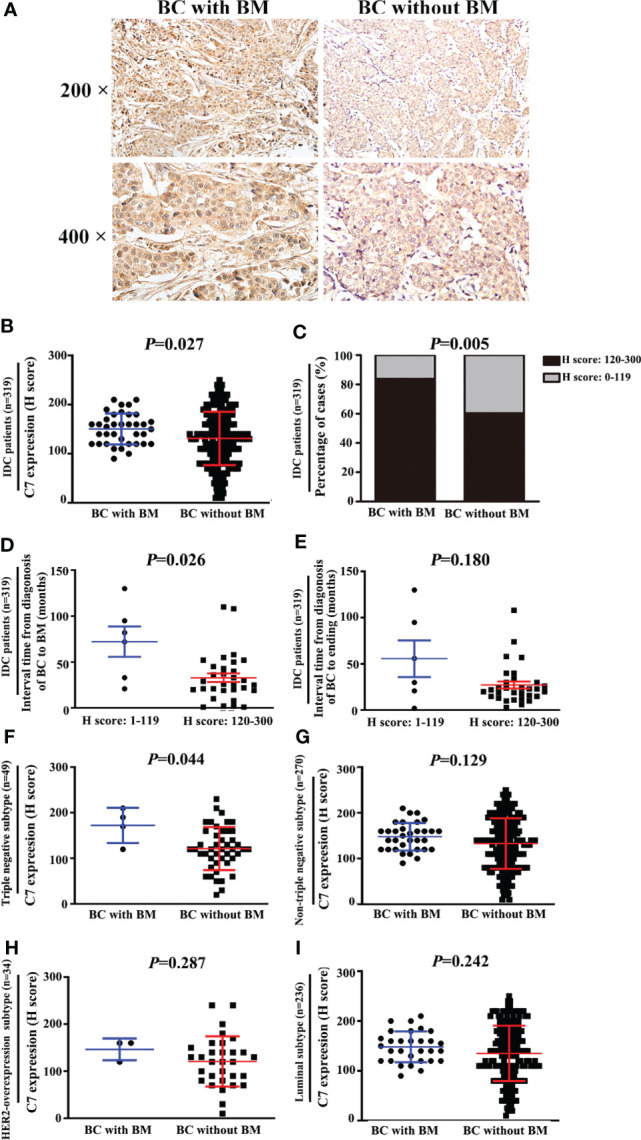
High expression of C7 promoted triple negative subtype patients developing bone metastasis (BM). **(A)** Representative images of C7 expression in primary tumor specimens from breast cancer patients with BM and patients without BM, respectively (magnification 200× and 400×). **(B)** C7 expression score in IDC patients who developed BM was higher than those without BM (*P* = 0.027). **(C)** Percentage of high C7 expression in patients who developed BM was higher than those than those without BM (*P*=0.005). **(D)** IDC patients with high expression of C7 exhibited earlier occurrence of BM. The median interval time from the diagnosis of breast cancer (BC) to BM in patients with high C7 expression was shorter than the low C7 expression group (*P* = 0.026). **(F)** In the triple negative subtype, C7 expression in patients with BM was much higher than that in those without BM (*P* = 0.004). **(G, H)** In the non-triple negative subtype **(F, G)**, HER2-overexpression subtype **(F)** and luminal B subtype **(G)**, no statistical difference of C7 expression was found between patients with BM and those without BM. **(I)** In luminal subtype, C7 expression in patients with BM was much higher than that in those without BM (*P* = 0.004). [**(B, D–I)**: Mann-Whitney U test, C: χ^2^ test].

In order to validate the relationship between C7 expression and breast cancer bone metastasis, the total 319 IDC patients were divided into two groups: 37 cases with bone metastasis and 282 cases without bone metastasis. C7 expression in breast cancer patients with bone metastasis (median H score: 155.0) was higher than those without bone metastasis (median H score: 130.0, **Figures 4A, B**). Percentage of high C7 expression in patients who developed BM was higher than those than those without BM (*P* = 0.005, **Figure 4C**). Furthermore, patients with a high C7 expression showed a shorter interval time (median 26.0 months) from their diagnosis of breast cancer to bone metastasis, compared with those with a low C7 expression (median 77.0 months, *P* = 0.026, **Figure 4D**). While there was no difference in the interval time between patients with a high C7 expression from their diagnosis of breast cancer to ending and those with a low C7 expression (*P* = 0.180, **Figure 4E**). Further analysis showed that in the triple negative subtype, patients with bone metastasis exhibited a higher C7 expression than those without bone metastasis (*P* = 0.044, **Figure 4F**). While, we did not find a similar trend in the non-triple negative subtype (*P* = 0.129, **Figure 4G**), HER2-overexpression subtype (*P* = 0.287, **Figure 4H**), or luminal B subtype (*P* = 0.242, **Figure 4I**).

### High Expression of C7 Indicated a Worse Prognosis of Patients Treated With Taxane and Anthracycline-Based Chemotherapy

**Figure 5:**
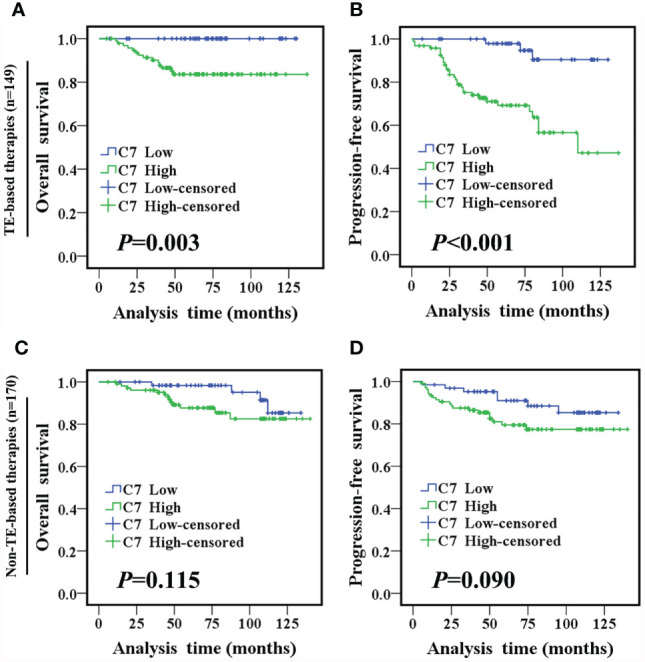
High C7 expression indicated a shorter survival in breast cancer patients treated with TE-based chemotherapy. **(A, B)** OS and PFS curves of IDC patients treated with TE-based chemotherapy (n = 149). **(C, D)** OS and PFS curves of IDC patients treated with non-TE-based chemotherapy (n = 170). [**(A–D)** log-rank test].

**Figure 6:**
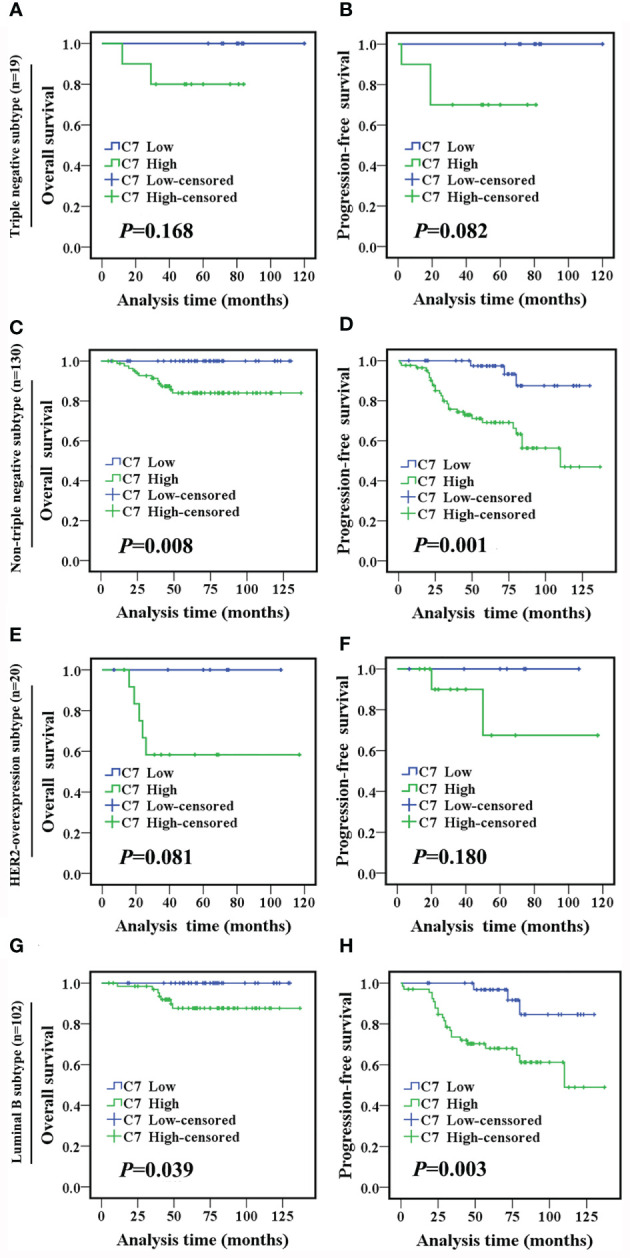
High C7 expression indicated a shorter survival in breast cancer patients treated with TE-based chemotherapy, especially in the luminal B subtype. **(A, B)** OS and PFS curves of triple negative subtype patients who received TE-based chemotherapy (n = 19). **(C, D)** OS and PFS curves of non-triple negative subtype patients who received TE-based chemotherapy (n = 130). **(E, F)** OS and PFS curves of the HER2-overexpression subtype patients who received TE-based chemotherapy (n = 20). **(G, H)** OS and PFS curves of luminal B subtype patients who received TE-based chemotherapy (n = 102). [**(A–H)**: log-rank test].

TE (taxane and anthracycline)-based chemotherapy is a part of the standard of care in the first line treatment of metastatic breast cancer. Then, we analyzed the relationship between C7 expression and prognosis of patients treated with conventional TE-based chemotherapy (n = 149), which were included in 319 IDC specimens. High expression of C7 indicated a shorter OS (*P* = 0.003, **Figure 5A**) and PFS (*P* < 0.001, **Figure 5B**) in patients treated with TE-based chemotherapy. However, no correlations between C7 expression and the OS (*P* = 0.115, **Figure 5C**) or PFS (*P* = 0.090, **Figure 5D**) were found in 170 patients who received non-TE-based chemotherapy. Afterwards, the 149 patients treated with TE-based chemotherapy were classified into subgroups according to the molecular subtypes. The survival analysis showed that C7 expression was not associated with the survival of patients in the triple negative subtype; while a high C7 expression indicated a poor survival in the Luminal B subtype (**Figures 6A–H**).

**Figure 7:**
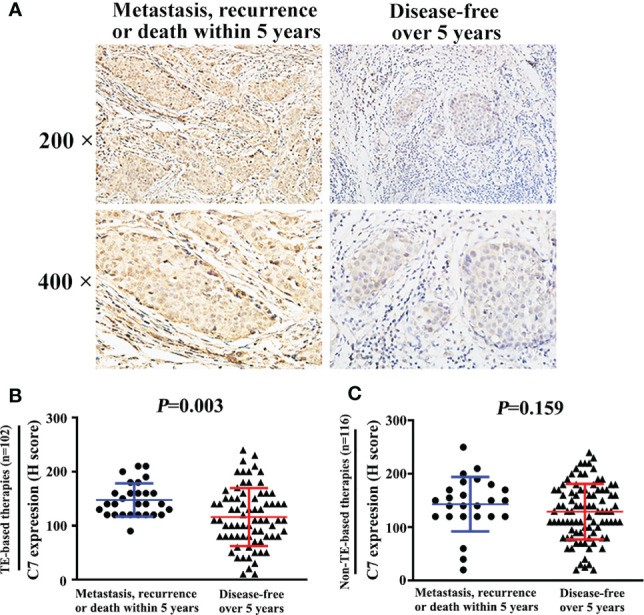
High expression of C7 promoted breast cancer progression in patients treated with TE-based chemotherapy. **(A)** Among 102 patients treated with TE-based chemotherapy, representative images of C7 expression in patients who developed metastasis, recurrence, or death within 5 years and patients who were disease-free over 5 years, respectively (magnification 200× and 400×). **(B)** Among TE-based chemotherapy-treated patients, C7 expression in patients who developed metastasis, recurrence, or death within 5 years was higher than those who were disease-free over 5 years. **(C)** Among non-TE-based chemotherapy-treated cases, no significant difference of C7 expression was found in patients who developed metastasis, recurrence, or death within 5 years and those who were disease-free over 5 years. [**(B, C)**: Mann-Whitney U test].

**Figure 8:**
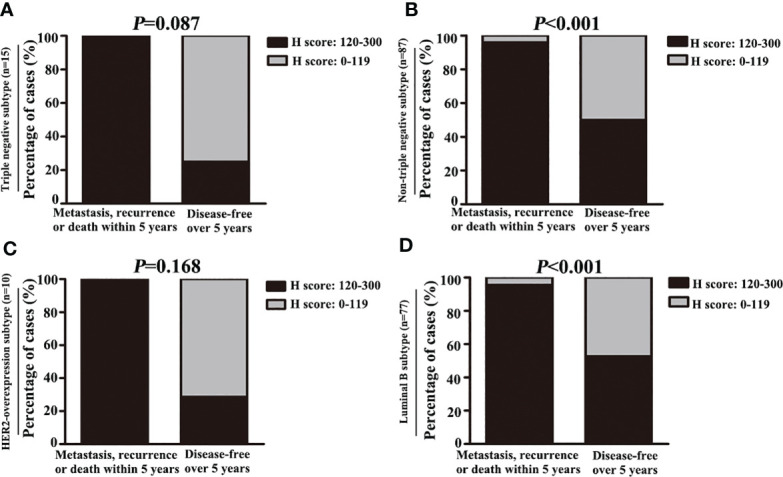
High expression of C7 promoted disease progression in luminal B subtype patients treated with TE-based chemotherapy. **(A, B)** Among patients treated with TE-based chemotherapy, the percentage of high C7 expression in patients who developed metastasis, recurrence, or death within 5 years was higher than those who were disease-free over 5 years in the non-triple negative subtype **(B)**, but not in the triple negative subtype **(A)**. **(C, D)** Among patients treated with TE-based chemotherapy, the percentage of high C7 expression in patients who developed metastasis, recurrence, or death within 5 years was higher than those who were disease-free over 5 years in the luminal B subtype **(D)**, but not in the HER2 overexpression subtype **(C)**. [**(A–D)**: χ^2^ test].

Moreover, among the 149 patients who received TE-based chemotherapy, patients who developed metastasis, recurrence, or death within 5 years exhibited a higher C7 expression (median H score: 140) than those who were disease-free over the same 5 years (median H score: 110, *P* = 0.003, **Figures 7A, B**). Consistently, among the patients treated with TE-based chemotherapy, patients who developed metastasis, recurrence, ordeath within 5 years exhibited a higher percentage of C7 highexpression than those who were disease-free over the same 5 years (**Table 5**). However, we did not find a similar trend in patients treated with non-TE-based chemotherapy (P=0.159, **Figure 7C**). Meanwhile, among the patients who received TE-based chemotherapy, patients who developed metastasis, recurrence, or death within 5 years exhibited a higher percentage of high C7 expression in the luminal B subtype (**Figures 8A–D**).

**Table 5:** Relationship between C7 expression and prognosis of IDC patients treated with TE chemotherapy.

	n	C7 score, n (%)		*r*_s_	*P*-value^a^
		Low (0–119)	High (120–300)		
**Metastasis, recurrence or death within 5 years**	**28**	**1 (3.6)**	**27 (96.4)**	**-0.459**	<0.001^***^
**Disease-free over 5 years**	**74**	**40 (54.1)**	**34 (45.9)**		

a P-value was calculated by Spearman’s Rank-Correlation test.

***P < 0.001.

### Breast Cancer Patients With High Expression of C7 Were Insensitive to Taxane and Anthracycline Neoadjuvant Chemotherapy

**Figure 9:**
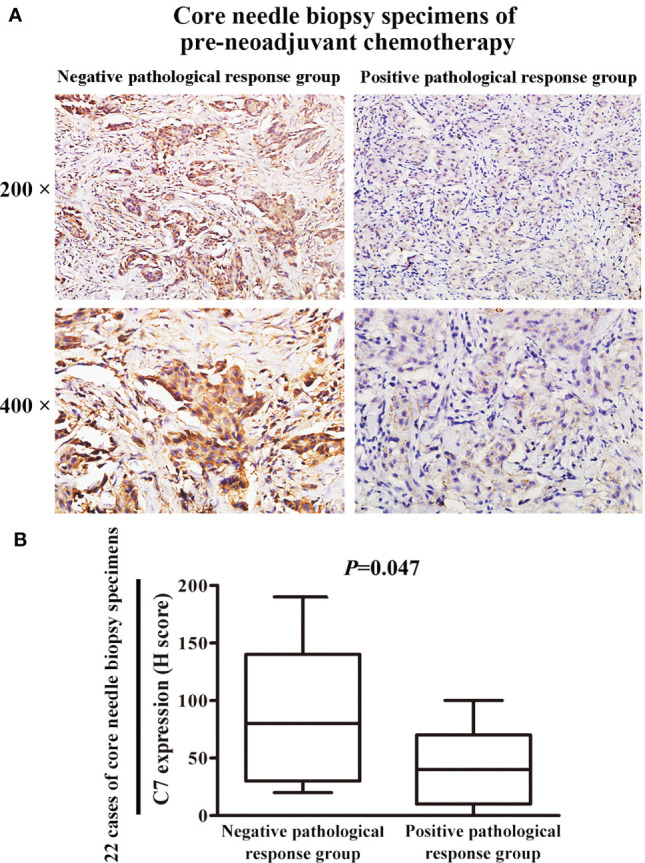
Patients with high C7 expression were insensitive to TE neoadjuvant chemotherapy. **(A)** Representative immunohistochemical images of C7 expression in both positive and negative pathological response groups, respectively (magnification 200× and 400×). **(B)** C7 expression in the negative pathological response group (n = 7) was higher than the positive pathological response group (n = 15, Mann-Whitney U test, *P* = 0.047).

Then, a cohort of patients (22 cases) treated with TE neoadjuvant chemotherapy was used to further confirm the relationship between C7 expression and TE-based chemosensitivity. The 22 patients were divided into two groups: positive pathological response group (15 cases) and negative pathological response group (7 cases). We found that C7 expression in the negative pathological response group (H score: 20 to 190, median: 80) was higher than that in the positive pathological response group (H score: 0–100, median: 40, *P* = 0.047, **Figures 9A, B**).

### Gene Set Enrichment Analysis of C7 in The Cancer Genome Atlas Database

To further gain insights into the mechanisms of the role of C7 in breast cancer progression, the analysis of the RNA-seq data of 817 breast cancer patients of the Cancer Genome Atlas (TCGA) was performed. Genes in high and low C7 expression patients were enriched by using the GSEA software for Kyoto Encyclopedia of Genes (KEGG) and Genomes pathway and Gene Ontology (GO) functional enrichment analysis. KEGG analysis suggested that changes were significantly enriched in the “VEGF signaling pathway”, “MAPK signaling pathway”, and “JAK stat signaling pathway” (**Supplementary Figure S2A**). GO analysis showed that changes in the biological process (BP) term were significantly enriched in “positive regulation of MAPK cascade”, “ERK1 and ERK2 cascade”, and “regulation of BMP signaling pathway” (**Supplementary Figure S2B**). It suggested that C7 may promote breast cancer progression by activating the VEGF, MAPK, or JAK stat signaling pathways, and also, C7 promoting breast cancer bone metastasis may be mediated by the bone morphogenetic protein (BMP) signaling pathway.

As well as known, anthracyclines interfere at the interface of Topo II-DNA with their sugar moieties and the cyclohexane ring A, which ultimately results in enzyme-mediated DNA damage in the form of double strand break (DSB) (14, 15). Taxane is a microtubule-stabilizing agent that impairs the proper assembly of mitotic spindles, leading to mitotic arrest and mis-segregation of chromosomes (16). GO analysis suggested that changes in the BP term were significantly enriched in DNA repair and microtubule associated process, such as “regulation DNA repair”, “recombination of DNA repair”, “positive regulation of DNA repair”, “double strand DNA repair”, “microtubule organizing center organization”, “microtubule organizing center localization”, “microtubule cytoskeleton organization involved in mitosis”, and “microtubule-based movement” (**Supplementary Figure S2B**). Changes in the cellular component (CC) term were significantly enriched in “site of damage”, “microtubule”, “microtubule associated complex”, and “DNA repair complex” (**Supplementary Figure S2C**). Changes in the molecular function (MF) term were also significantly enriched in “microtubule motor activity”, “microtubule binding” “ATP dependent microtubule mote activity”, and “DNA replication origin binding” (**Supplementary Figure S2D**). These results revealed that C7 may regulate the sensitivity of TE-based chemotherapy by affecting DNA repair and microtubule associated process.

## Discussion

Our study investigated the clinical and prognostic effects of C7 in breast cancer for the first time. C7 expression of IDC tissues was higher than non-neoplastic tissues adjacent to tumor and DCIS. Moreover, C7 was an independent prognosis factor and a high expression of C7 indicated a poor prognosis of IDC patients. These observations indicated that C7 may act as a tumor promoter, consistent with the study by Saijoh about the role of C7 in ovarian cancer (11). Our further study showed that a high expression of C7 promoted breast cancer bone metastasis. Firstly, we noticed that there was a weak association between C7 and bone metastasis, but there was no difference between C7 expression and lung metastasis, liver metastasis, or brain metastasis (**Table 4**). In fact, the most common site of breast cancer metastasis is the bone, occurring in about 70% of patients with metastatic breast cancer (17, 18). Consistently, in our cohort, patients with bone metastasis were more than those with lung metastasis, liver metastasis, and brain metastasis, respectively. In order to validate the relationship between C7 expression and breast cancer bone metastasis, the total 319 IDC patients were divided into two groups: 37 cases with bone metastasis and 282 cases without bone metastasis. C7 expression in breast cancer patients with bone metastasis was higher than those without bone metastasis (**Figures 4A, B**). Furthermore, patients with a high C7 expression showed a shorter interval time from their diagnosis of breast cancer to bone metastasis, compared with those with a low C7 expression (**Figure 4C**). Altogether, these results suggested that a high expression of C7 promoted breast cancer bone metastasis, but further *in vitro* and *in vivo* investigations are needed to confirm these findings.

Although emerging evidence showed the role of C7 in several malignances, little research focused on the mechanism of C7 function in tumor progression. In our present study, KEGG and GO enrichment analysis suggested that C7 may promote breast cancer progression by activating the VEGF, MAPK, or JAK stat signaling pathways. According to these clues, we will perform *in vitro* and *in vivo* assays to confirm the exact mechanism of C7 in breast cancer progression in the further study.

In fact, C7, as a single molecule, may play limited roles in tumor progression. Various studies showed that the membrane attack complex, which is composed of C5b–9 (C5b, C6, C7, C8, C9), could induce the activation of several tumorigenesis signal transduction pathways, including the MAPK family, PKC signaling, Gi protein/PI3K/Akt pathway, and Ras/Raf/ERK1 pathway (19–21). In addition, the sublytic effects of C5b-9 involved cell cycle activation, accomplished by affecting main cell cycle kinases and regulators, such as CDK4, CDK2, p21, CDC2, cyclin D1, and PCNA (22, 23). Moreover, sublytic C5b–9 had an antiapoptotic effect by regulating the phosphorylation of FOXO1 and Bad, and inhibiting the activation of Bid, caspase-8, and NF-κB (24–27). Therefore, it should be better to detect other components, such as C5b, C6, C8, and C9, to provide a more comprehensive information to reveal the roles of the complement in breast cancer progression.

TE-based chemotherapy is a part of the standard of care in the first line treatment of metastatic breast cancer and its clinical use is widespread (28). However, only about 15% patients could achieve pathologic complete response. Therefore, a more detailed classification is necessary to screen a more suitable population to TE chemotherapy (29). A previous study reported that the level of complement C3 α1 (an isoform of cleaved C3 α-chain and a complement activation marker) in breast cancer patients was increased in TE-chemotherapy responders compared with non-responders. The possible explanation may be TE-based chemotherapy induced tumor cells apoptosis to activate the complement system (30). Although the main complement system activation pathways generate C3 convertases efficiently cleaving C3 into C3a and C3b, killing targeted cells finally requires the terminal C5b-9 MAC (membrane attack complex) (31). Therefore, detecting the level of MAC components in breast cancer patients is more reasonable to predict the chemosensitivity.

## Conclusions

Taken together, our study provided the first evidence that C7 expression was an independent prognosis factor in IDC patients. High expression of C7 indicated poor prognosis, especially in the triple negative subtype and luminal B subtype. C7 high expression promoted breast cancer to develop bone-specific metastasis, mainly in the triple negative subtype. Furthermore, patients with high C7 expression were insensitive to TE-based chemotherapy. These findings highlight the importance of C7 in breast cancer progression and lay a foundation to help clinicians improve the identification of patients for TE chemotherapy by C7 in the era of precision medicine.

## Data Availability Statement

The raw data supporting the conclusions of this article will be made available by the authors, without undue reservation.

## Ethics Statement

This study was approved by the Institutional Ethics Committee of Tianjin Medical University Cancer Institute and Hospital. The patients/participants provided their written informed consent to participate in this study. Written informed consent was obtained from the individual(s) for the publication of any potentially identifiable images or data included in this article.

## Author Contributions

HZ performed the data analysis, prepared the figures, and wrote the manuscript. YZ contributed to the immunohistochemistry experiments. XL performed the Western blot experiments. LF and FG collected the patient samples and interpreted the data. YM designed the research and wrote the manuscript. All authors contributed to the article and approved the submitted version.

## Funding

This work was supported by the Scientific Research Program of Tianjin Municipal Education Commission from HZ (2020KJ130).

## Conflict of Interest

The authors declare that the research was conducted in the absence of any commercial or financial relationships that could be construed as a potential conflict of interest.

## Publisher’s Note

All claims expressed in this article are solely those of the authors and do not necessarily represent those of their affiliated organizations, or those of the publisher, the editors and the reviewers. Any product that may be evaluated in this article, or claim that may be made by its manufacturer, is not guaranteed or endorsed by the publisher.

## Abbreviations

DAB, 3,3’-diaminobenzidine tetrahydrochloride; DCIS, ductal carcinoma *in situ*; IDC, invasive ductal carcinoma; IHC, Immunohistochemistry; OS, overall survival; PFS, progression-free survival; S-P, streptavidin-peroxidase; TE, taxane and anthracycline.
