# Supplementary figures and images for: High Expression of Complement Component C7 Indicates Poor Prognosis of Breast Cancer and Is Insensitive to Taxane-Anthracycline Chemotherapy
Source: Front Oncol. 2021 Sep 24;11:724250. doi: 10.3389/fonc.2021.724250 (PMC8497743; doi:10.3389/fonc.2021.724250)

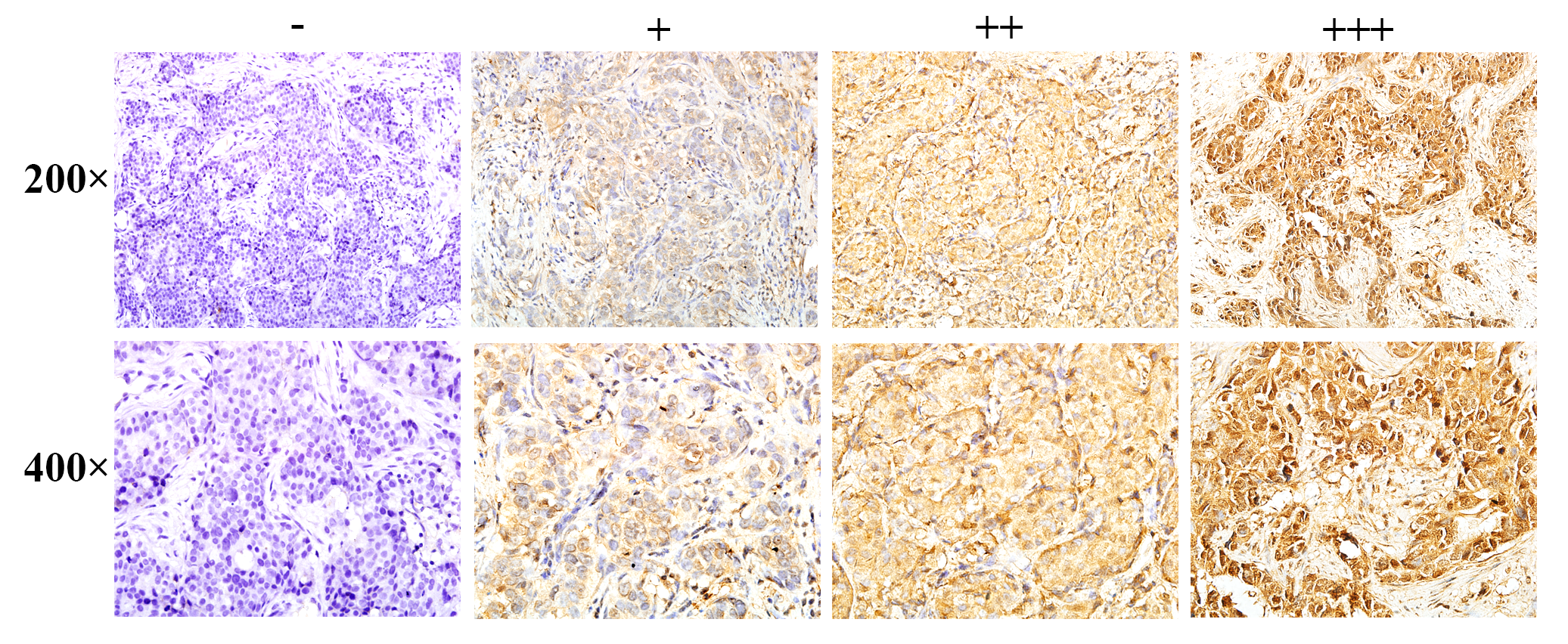

Supplement: Supplementary file 1 [file Image_1.tif]

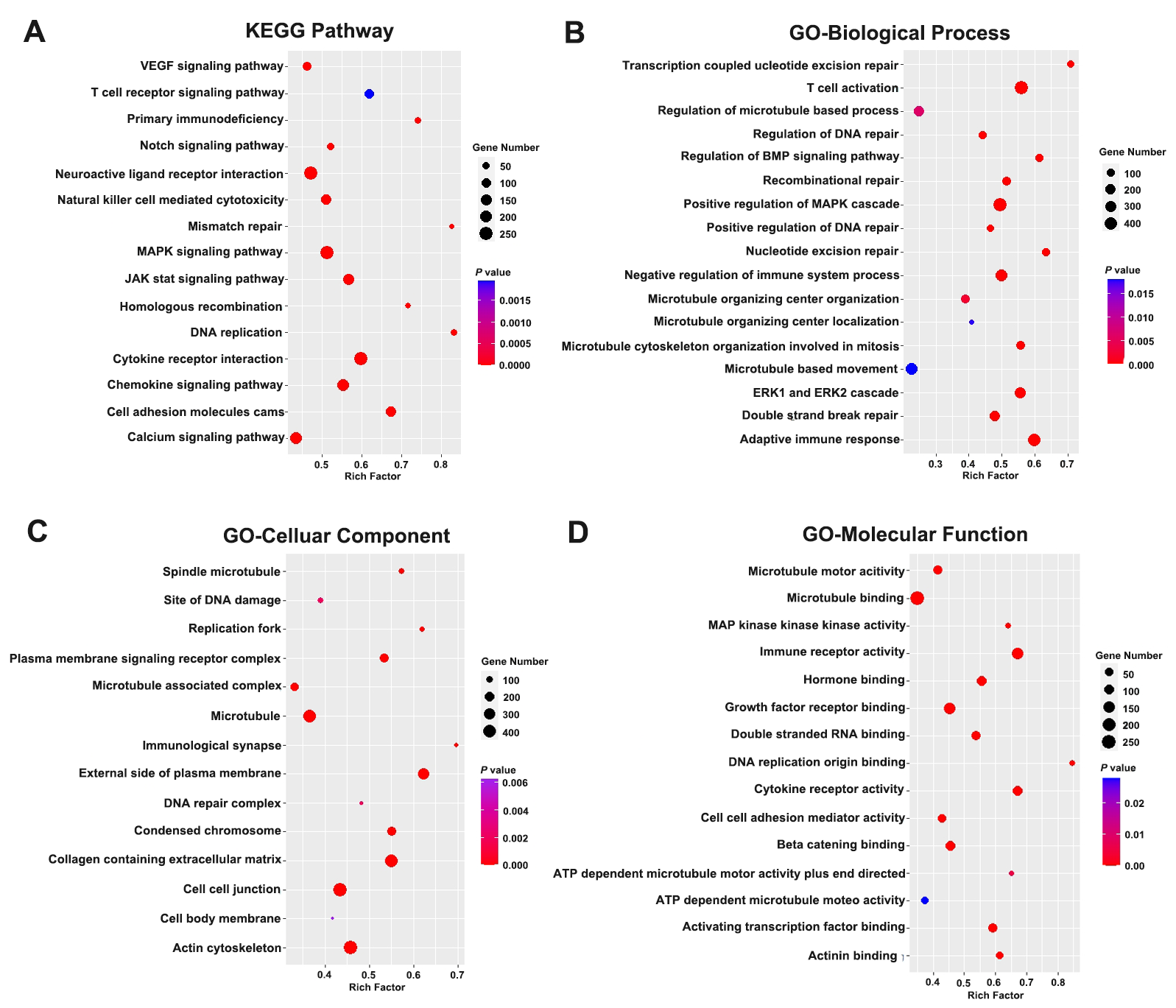

Supplement: Supplementary file 2 [file Image_2.tif]
