# Supplementary material for: High Expression of Complement Component C7 Indicates Poor Prognosis of Breast Cancer and Is Insensitive to Taxane-Anthracycline Chemotherapy
Source: Front Oncol. 2021 Sep 24;11:724250. doi: 10.3389/fonc.2021.724250 (PMC8497743; doi:10.3389/fonc.2021.724250)
Supplement: Supplementary file 3 [file Table_1.docx]

**Supplementary Table S1. Demographic data of IDC patients (n=331).**

| **Pathological features** | **Year** | | | | | |
| --- | --- | --- | --- | --- | --- | --- |
|  | **2004** | **2005** | **2006** | **2007** | **2008** | **2009** |
| **n** | **10** | **178** | **43** | **20** | **52** | **28** |
| **Age, year** |  |  |  |  |  |  |
| **<50** | **6** | **94** | **27** | **6** | **30** | **11** |
| **≥50** | **4** | **84** | **16** | **14** | **22** | **17** |
| **cTNM stage^a^** |  |  |  |  |  |  |
| **Ⅰ** | **3** | **27** | **6** | **3** | **11** | **4** |
| **Ⅱ** | **4** | **118** | **29** | **12** | **35** | **16** |
| **Ⅲ-Ⅳ** | **3** | **33** | **8** | **5** | **5** | **8** |
| **Histological grade** |  |  |  |  |  |  |
| **Ⅰ** | **0** | **3** | **0** | **1** | **2** | **0** |
| **Ⅱ** | **8** | **140** | **35** | **16** | **37** | **24** |
| **Ⅲ** | **2** | **35** | **8** | **3** | **13** | **4** |
| **Tumor size, cm** |  |  |  |  |  |  |
| **<2** | **0** | **13** | **5** | **0** | **3** | **5** |
| **2-5** | **8** | **138** | **32** | **12** | **43** | **20** |
| **>5** | **2** | **27** | **6** | **8** | **6** | **3** |
| **Lymphnode metastasis** |  |  |  |  |  |  |
| **0** | **2** | **65** | **14** | **3** | **32** | **8** |
| **1-3** | **3** | **48** | **15** | **2** | **8** | **6** |
| **4-9** | **1** | **29** | **3** | **8** | **5** | **7** |
| **>9** | **4** | **36** | **11** | **7** | **7** | **7** |
| **Distant metastasis^a^** |  |  |  |  |  |  |
| **No** | **7** | **139** | **36** | **14** | **43** | **20** |
| **Yes** | **2** | **35** | **5** | **6** | **8** | **4** |
| **ER status ^b^** |  |  |  |  |  |  |
| **Negative** | **6** | **62** | **19** | **6** | **20** | **14** |
| **Positive** | **4** | **116** | **24** | **14** | **32** | **14** |
| **PR status^c^** |  |  |  |  |  |  |
| **Negative** | **5** | **53** | **11** | **4** | **30** | **20** |
| **Positive** | **5** | **125** | **32** | **16** | **22** | **8** |
| **HER2 stauts ^d^** |  |  |  |  |  |  |
| **Negative** | **8** | **124** | **28** | **11** | **30** | **18** |
| **Positive** | **2** | **54** | **15** | **9** | **22** | **10** |
| **Ki-67 status** |  |  |  |  |  |  |
| **Negative** | **10** | **18** | **2** | **1** | **10** | **4** |
| **Positive** | **0** | **160** | **41** | **19** | **42** | **24** |

**^a^ some missing data.**

**^b^ ER status: estrogen receptor status.**

**^c^ PR status: progesterone receptor status.**

**^d^ HER2 status: human epidermal growth factor receptor-2 status.**
