# Supplementary material for: High Expression of Complement Component C7 Indicates Poor Prognosis of Breast Cancer and Is Insensitive to Taxane-Anthracycline Chemotherapy
Source: Front Oncol. 2021 Sep 24;11:724250. doi: 10.3389/fonc.2021.724250 (PMC8497743; doi:10.3389/fonc.2021.724250)
Supplement: Supplementary file 4 [file Table_2.docx]

**Supplementary Table S2. Demographic data of subgroups in IDC patients.**

| **Subgroups** | **n** | **C7 score, n** | **C7 score, n** |
| --- | --- | --- | --- |
|  |  | **Low(0-119)** | **High(120-300)** |
| **Triple negative subtype** | **52** | **22** | **30** |
| **TE-based therapy** | **20** | **10** | **10** |
| **Non-TE-based therapy** | **32** | **12** | **20** |
| **Non-triple negative subtype** | **279** | **100** | **179** |
| **TE-based therapy** | **133** | **46** | **87** |
| **Non-TE-based therapy** | **146** | **54** | **92** |
| **HER2-overexpression subtype** | **35** | **13** | **22** |
| **TE-based therapy** | **20** | **7** | **13** |
| **Non-TE-based therapy** | **15** | **6** | **9** |
| **Luminal subtype** | **244** | **87** | **157** |
| **TE-based therapy** | **113** | **38** | **75** |
| **Non-TE-based therapy** | **131** | **49** | **82** |
| **Luminal A subtype** | **30** | **10** | **20** |
| **TE-based therapy** | **8** | **3** | **5** |
| **Non-TE-based therapy** | **22** | **7** | **15** |
| **Luminal B subtype** | **214** | **77** | **137** |
| **TE-based therapy** | **105** | **35** | **70** |
| **Non-TE-based therapy** | **109** | **42** | **67** |
